# High Dynamic Range Spectral Imaging Pipeline For Multispectral Filter Array Cameras

**DOI:** 10.3390/s17061281

**Published:** 2017-06-03

**Authors:** Pierre-Jean Lapray, Jean-Baptiste Thomas, Pierre Gouton

**Affiliations:** 1 MIPS Laboratory, Université de Haute Alsace, 68093 Mulhouse, France; pierre-jean.lapray@uha.fr; 2 The Norwegian Colour and Visual Computing Laboratory, NTNU-Norwegian University of Science and Technology, 2815 Gjøvik, Norway; 3 Le2i Laboratory, FRE CNRS 2005, Université de Bourgogne Franche-Comté, 21000 Dijon, France; pgouton@u-bourgogne.fr

**Keywords:** spectral imaging, spectral filter arrays, high dynamic range, image database

## Abstract

Spectral filter arrays imaging exhibits a strong similarity with color filter arrays. This permits us to embed this technology in practical vision systems with little adaptation of the existing solutions. In this communication, we define an imaging pipeline that permits high dynamic range (HDR)-spectral imaging, which is extended from color filter arrays. We propose an implementation of this pipeline on a prototype sensor and evaluate the quality of our implementation results on real data with objective metrics and visual examples. We demonstrate that we reduce noise, and, in particular we solve the problem of noise generated by the lack of energy balance. Data are provided to the community in an image database for further research.

## 1. Introduction

Spectral filter arrays (SFA) technology [1] provides a compact and affordable means to acquire multispectral images (MSI). Such images have been proven to be useful in countless applications, but there extended use to general computer vision was limited due to complexity of imaging set-up, calibration and specific imaging pipelines and processing. In addition, spectral video are not easily handled either. SFA, however, developed around a very similar imaging pipeline to color filter arrays (CFA), which is rather well understood and already implemented in many solutions. Indeed, SFA, similarly to CFA, is a spatio-spectral sampling of the scene captured by a single shot of a solid-state, single, image sensor. In this sense, SFA may provide a conceptual solution that improves vision systems by trading spatial resolution for more spectral information.

Until recently, only simulations of SFA cameras were available, which made its experimental evaluation and validation difficult. Recent works on optical filters [2,3,4] in parallel to the development of SFA camera prototypes in the visible electromagnetic range [5], in the near infrared (NIR) [6] and in combined visible and NIR [7,8] permitted the commercialization of solutions, e.g., Imec [9], Silios [10], Pixelteq [11]. In addition, several color cameras include custom filter arrays that are in-between CFA and SFA (e.g., [12,13]). Recent applications in medical imaging [14], agriculture and environment [15] have been published. This indicates that we could consider the application of this technology to large scale use soon after the development of standard imaging pipelines and drivers.

We define and demonstrate the imaging pipeline in this communication. One strong remaining limitation of SFA is to preserve the energy balance between channels [16,17] while capturing a scene. Indeed, due to the large number of filters and their spectral characteristics, i.e., narrow band sensitivities and inadequacy with the scene and illumination, or large inhomogeneity between filter shapes, it is frequent to observe one or several channels under- or over-exposed for a given integration time and illumination, which is common to all filters. This may be solved in theory, by optimizing the filters before to create the sensor [17]. But filter realization is not yet very flexible. Another way to solve this issue would be to develop sensors with per-pixel integration control. This is in development within some 3D silicon sensor concepts [18,19], but this technology is at its very beginning, despite recent advances.

On the other hand, in gray-level and color imaging, the problem of under and over-exposure of parts of the scene is addressed by means of high dynamic range (HDR) imaging [20,21]. HDR imaging permits to potentially recover the radiance of the scene independently of the range of intensities present in the scene. Since the dynamic range of a given sensor is limited, the quantization of the radiance values is a source of problems. The signal detection of very low intensity is limited by the dark noise. On the other hand, high intensities of the input signal cannot be completely recovered and are sometimes voluntarily ignored (saturated pixels). To overcome these problems, a low exposure image could be used to quantify the highest intensities, whereas a high exposure allows us to quantify relatively low light signals well. Such an approach may also be used to bring less and more sensitive channels to a common representation space with a reduced noise amount.

In an ideal configuration, an HDR image is created by bringing standard dynamic range (SDR, typically 8 bits per channel) images in the same domain by dividing each image by its particular exposure time, and then by summing the corresponding pixel values. However, due to the effect of electronic circuits, most of the cameras have a non-linear processing regarding to the digitization of intensities, leading to a finite pixel brightness range and definition. This non-linear transformation is materialized by the camera response function, denoted g(i), where *i* indexes the pixel value. It is assumed that this curve is monotonic and smooth. Some algorithms have been developed to recover this characteristic [21,22,23]. The most common method is the non-parametric technique from Debevec and Malik [21]. For a given exposure time and camera pixel value, the relative radiance value is estimated by using the integration times, g(i) and a weighting function ω(i). Debevec and Malik use a “hat” function as weighting function, based on the assumption that mid-range pixels (values close to 128 for 8-bits sensors) are the most reliable and the best exposed pixel for a given scene and integration time. In addition, recent advances have been done on the capture and processing of HDR video with low latency, using hardware-based platform [24,25,26]. For HDR video, merging images captured at different sequential moments could lead to ghost artifacts when there are moving objects. This has been largely studied in recent years [27,28]. So, we argue that such methodology could be embedded in the SFA imaging pipeline without breaking the advantages of SFA technology for computer vision.

HDR multispectral acquisition is already treated by, e.g., Brauers et al. [29] and Simon [30]. However, they consider the problem of HDR using individual bands acquired sequentially, so each band is treated independently for potentially different integration times. In the case of SFA, we may consider specific joined processes. We communicated preliminary qualitative results at the Scandinavian Conference on Image Analysis (SCIA 2017) [31], and this paper extends, generalizes, and evaluates widely our preliminary results. We compare the HDR resulting images with an SDR database of the same objects [32]. In addition, we make our HDR database available for further research as supplementary material.

In this paper, we first generalize the imaging CFA pipeline to SFA in Section 2. This new SFA imaging pipeline embeds the HDR concept; It is based on multiple exposure spectral raw images. Then, the experimental implementation is developed in Section 3, the implementation is based on real data acquired with a prototype state-of-the-art camera [8] that captures visible and NIR information. Description of the database of images are provided in Section 4. Results and analysis are based on objective metric scores and visual examples in Section 5.

## 2. Imaging Pipelines

### 2.1. CFA Imaging Pipeline

**Figure 1:**
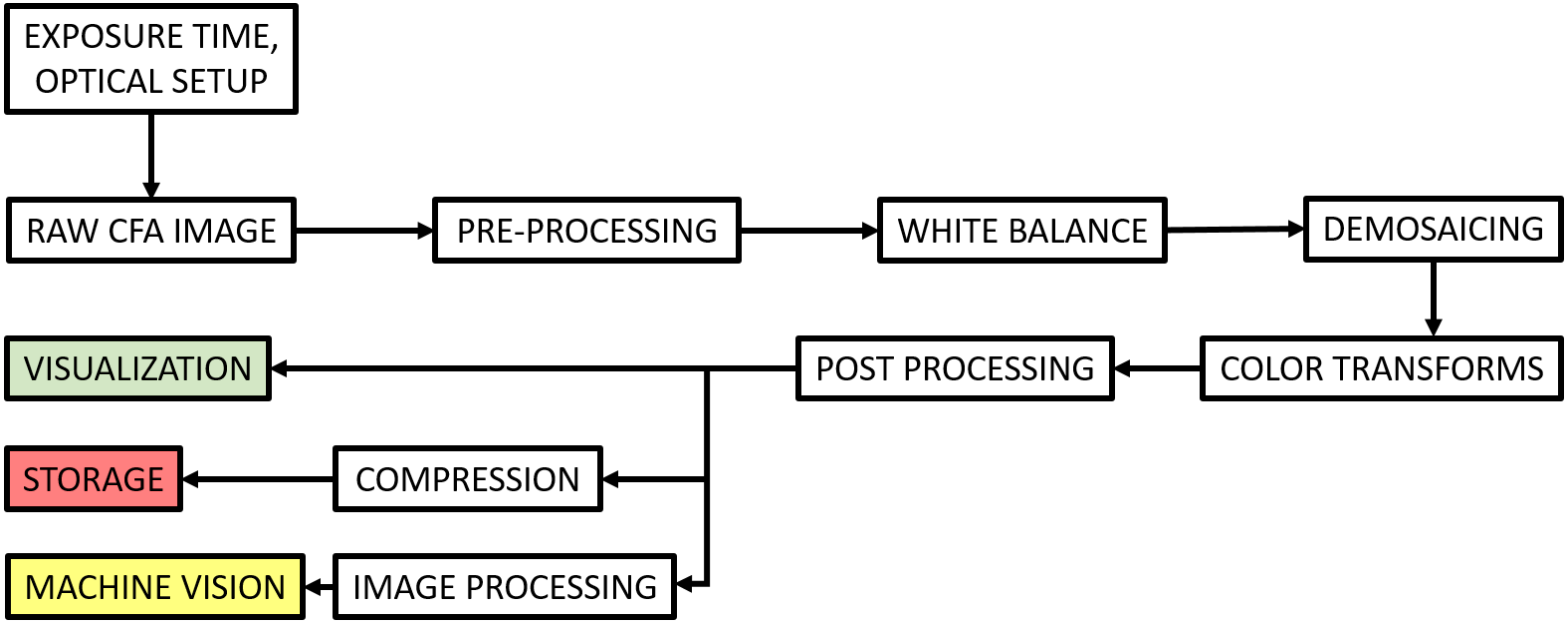
Color filter arrays (CFA) imaging pipeline similarly defined as in [37]. The pipeline contains pre-processing on raw data, which include for instance a dark noise correction and other denoising. Raw data would be corrected for illumination before to be demosaiced. Images are then projected into an adequate color space representation and followed by some post-processing, e.g., image enhancement, before coming out of the pipeline on a visualization media. Alternatively, this information could be compressed before archiving or be used for machine vision through adequate image processing.

Several CFA imaging pipelines exist. We can classify them in two large groups: one concerns the hardware and real-time processing community [33,34,35], the other concerns the imaging community [36,37,38]. A very general distinction is that the former one often considers the problem from the sensor and signal point of view and demosaics the raw image in very early steps, rather the latter considers the problem from a visualization point of view, and demosaics after or jointly with other processing such as white balance. In this work, we design the pipeline after the generic version defined by Ramanath et al. [37], which is shown in Figure 1.

### 2.2. HDR-CFA Imaging Pipeline

HDR imaging has been developed mostly within monochromatic sensors for the acquisition of HDR data. Indeed, the HDR capture is mostly an intensity process performed by channel [30]. However, there is a huge amount of work that developed the tone-mapping of HDR color images for visualization, (e.g., [39,40]).

**Figure 2:**
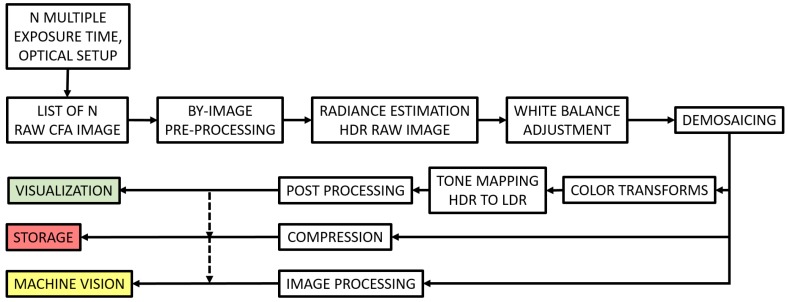
High dynamic range (HDR)-color filter arrays (CFA) imaging pipeline. In this case, the pre-processing is typically performed per image, similarly to the standard dynamic range (SDR)-CFA case. Then, radiance estimation is performed based on the multiple images, providing radiance raw images. White balancing and demosaicing are performed on this data. Then, the HDR image may be used as is for machine vision, or it continues into a visualization pipeline, where color transform, tone-mapping and image-enhancement may be applied before visualization. Bridge between the different output may occur if, for instance, the machine vision is designed to SDR content.

We encapsulate a general HDR imaging process in the previous pipeline such as shown in Figure 2. This pipeline is based on sequence of images of the same scene having different integration times. The HDR pipeline may have distinguished outputs: one leads to HDR radiance images, which can be stored or used for automatic application. Another leads to a display-friendly visualization of color images. Note that these two outputs may overlap in specific applications.

### 2.3. SFA Imaging Pipeline

**Figure 3:**
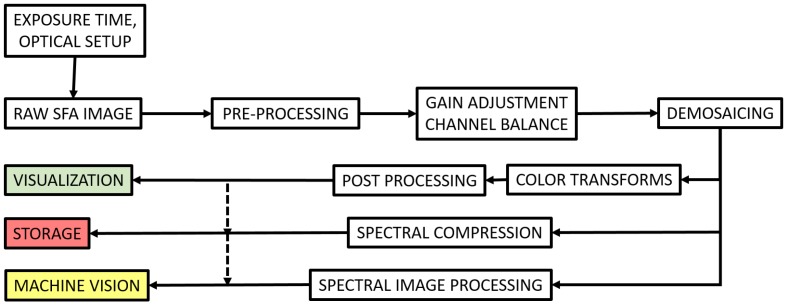
SFA imaging pipeline. At the instar of CFA, this pipeline defines some illumination discarding process and demosaicing. The spectral image would be typically used for application after demosaicing. However, these data may not be observable as they are, so the pipeline is prolonged for visualization. The color transform is ought to be slightly different than CFAs, for several channels are present and out of the visible range information, NIR, may be present in the spectral image. Compression of spectral data and spectral image processing, for, e.g., material identification or texture classification, are yet active research fields.

SFA sensors are currently investigated and developed, however beside demosaicing and application dedicated processing, the rest of the pipeline is not very well defined, thus understood, to our knowledge. We argue that a similar pipeline than CFA may be considered, which is then defined in Figure 3. In this pipeline, we still consider pre-processing as denoising and an equivalent to white balance as gain adjustment channel balance (referred to as multispectral constancy by some research). In the visualization pipeline, the color transform shall project the multispectral data into a color space representation.

### 2.4. HDR-SFA Imaging Pipeline

**Figure 4:**
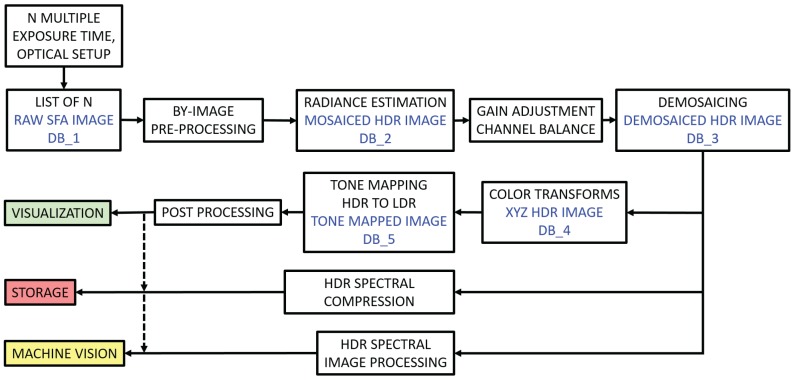
Our HDR-SFA imaging pipeline. The radiance estimation is performed on the list of raw image taken as a whole ($DB_{1}$ in the database), which permit to create the HDR raw images ($DB_{2}$ in the database). The raw HDR image may be corrected for illumination [41,42] and demosaiced by state-of-the-art methods ($DB_{3}$ in the database). After this, a visualization process projects the data into a HDR color representation CIEXYZ ($DB_{4}$ in the database), which is tone-mapped ($DB_{5}$ in the database) and processed for visualization on SDR media. Other outputs of the pipeline may be considered similarly to the previous pipelines.

According to the introductory discussion, we propose to extend the SFA pipeline to an HDR version. Beside the advantage of increasing the dynamic range of our images, we are also particularly interested in a better balance between channel sensitivities and to the reduction and an homogeneous distribution of noise by channel thanks to the increase of information. We propose to consider the raw SFA image as a gray-level image for relative radiance estimation, since this process is essentially a per-pixel operation. Thus, we perform all radiance reconstruction prior to any separation between bands. The pipeline is defined in Figure 4. We insist on the fact that this is only one possibility to consider the HDR pipeline for SFA, which has the advantage to imply only little modifications of the gray-level HDR pipeline and to permit the embedding of any individual algorithms in any of the boxes.

## 3. Implementation of the HDR-SFA Imaging Pipeline

This section explicitly defines what processing is embedded in each of the pipeline box in our experiment. We selected well-established and understood methods from the state-of-the-art in order to provide benchmarking proposal and analysis and not go towards the evaluation of each of those methods individually. Those methods are combined into the pipeline. Our proposal is not exclusive in the sense that any method may be used and different order of processing or joint processing may also be considered in the future.

The prototype SFA camera from Thomas et al. [8] is used in this study. Detailed information on sensitivities, spatial arrangement and other aspects may be found in their article. The raw images are pre-processed and denoised according to what is performed in their article, which is essentially a dark noise removal. Then, following the pipeline, HDR data are computed. Section 3.1 covers the HDR radiance estimation. HDR images are balanced according to each channel and illumination and demosaiced, according to Miao et al. [43] algorithm, which form the full resolution HDR multispectral images. The output of the pipeline and the visualization procedure are developed in Section 3.2. Discussion on the role of illumination is provided in Section 3.2.1.

### 3.1. HDR Generation

**Figure 5:**
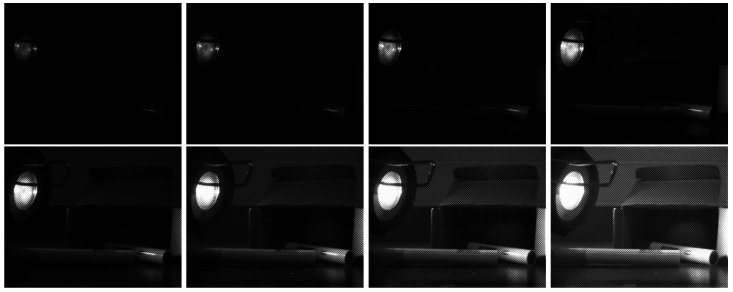
The set of SDR raw mosaiced images acquired with different exposure times: {0.125,0.22,0.5,1,2,4,8,16} ms (all spaced by one stop). These exposures are used to compute the global response curves of the prototype camera, shown in Figure 6a.

Debevec and Malik radiance reconstruction [21] is probably the most understood HDR imaging pipeline. The model is based on the assumption that pixel values can be related to the physical quantity of radiance, by using a computed camera response function, which is recovered through a self calibration method. Due to the digitization process that converts radiance into pixel value in the image, this mapping is generally nonlinear and a calibration should be done before any estimation. To reconstruct HDR images, the camera response function (CRF) must be estimated. We captured 8 SDR bracketed images at different exposure times, from 0.125 ms to 16 ms with a one-stop increment, see Figure 5. This leads to a good response curve estimation in term of robustness to noise.

**Figure 6:**
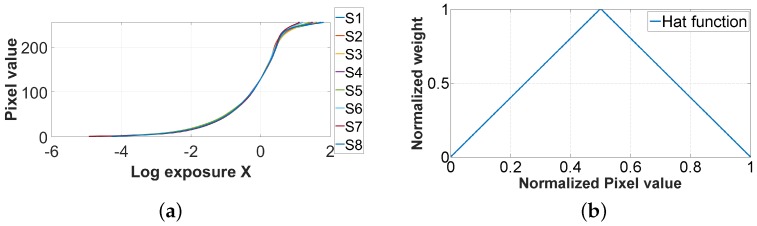
(**a**) Camera response functions to correct for the non-linearity between relative real radiance values and pixel intensities in the images. It is recovered from a complete image set shown in Figure 5. In the pipeline, the median of these curves is used to treat all of the pixels, independently of their spectral sensitivities; (**b**) The well-exposedness hat function used in this implementation.

The algorithm to recover the CRF is based on the resolution of a set of linear equations by the singular value decomposition method. The algorithm is generally applied on RGB cameras, and it recovers 3 different response curves, one by channel. In our case, as we get eight spectral channels, we recover eight curves (see Figure 6a). We notice that the dispersion is relatively low between each of the channels, so in the following, we use the median of these curves for all channels, allowing us to work directly on the raw data at once to generate HDR values.

As described in the pipeline, we recover the relative radiance values directly from the preprocessed raw data (mosaiced data). A number of 3 exposure times is selected. We chose only three exposures because it is a number commonly used in the literature [24,28], as it gives relatively high dynamic range and not too much ghost effects in case of video capture. The radiance values are recovered using the CRF, and by combining the pixel value with its corresponding exposure time [21]. A weighted sum of the radiance values computed from all exposure times is done using the hat weighting function, that gives more contribution to mid-range intensity pixels during the HDR reconstruction (see Figure 6b).

**Figure 7:**
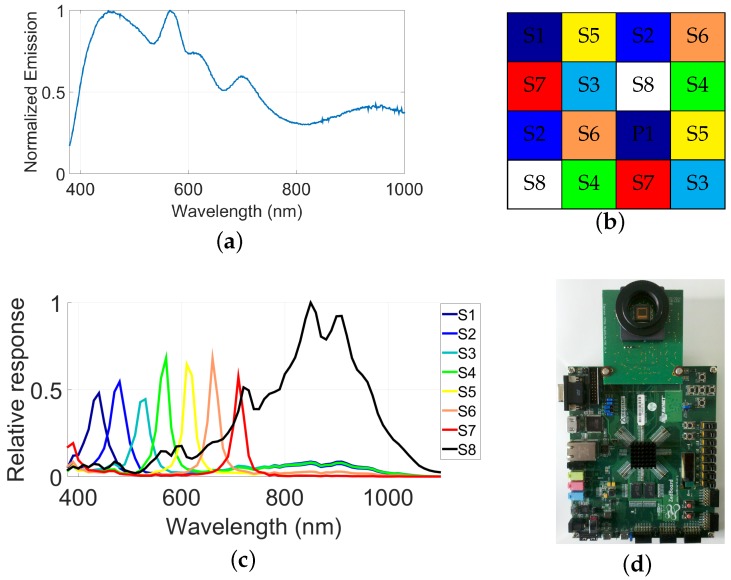
The hardware and acquisition procedure, from the illuminant source to the digitized output of the camera. (**a**) D65 simulator emission spectra used during the experiment; (**b**) Spatial distribution of filters over the sensor; (**c**) Joint spectral characteristics of optical filters and CMOS sensor [8]; (**d**) Camera and electronic architecture, composed of a FPGA (Field-Programmable Gate Array) board and an attached daughter card holding the SFA sensor.

After the radiance is estimated by pixel, we apply a balance that compensates for the different spectral sensitivities of each band i∈Nandi∈[1,8] and for the illumination spectral power distribution. We implement a linear correction similarly to a white balance in color camera based on Equation (1).

(1) $$\rho_{i}=\int_{400}^{1100}I_{Ill}\left(\lambda \right).S_{i}\left(\lambda \right)d\lambda ,\;$$

where $I_{Ill}\left(\lambda \right)$ is the spectral emission of the illuminant used, $S_{i}\left(\lambda \right)$ is the measured camera spectral sensitivity of each channel (see Figure 7c). We then can compute the multiplication gain factors *F* to correct the data and balance their energy as in Equation (2).

(2) $$F_{i}=\frac{\max\{\rho_{1},\ldots ,\rho_{8}\}}{\rho_{i}}\;$$

We obtain 8 factors $F_{S_{1}-S_{8}}=\left\{3.15,2.86,3.29,2.90,4.34,5.39,6.41,1.00\right\}$ that are applied to each of the channels. This can be performed independently of the illumination by removing its contribution in Equation (1). In this case, illuminant compensation would not be taken into account and should be handled in another process.

The raw HDR image is then demosaiced to recover the spatial resolution of each of the HDR spectral channels. We then obtain the HDR multispectral image.

### 3.2. Visualization and Other Output

Visualization is the traditional use of HDR data. To this end, we project the radiance data into an HDR coded CIEXYZ color space according to a linear color transform computed on the 24 Macbeth ColorChecker reflectance patches, and the scene acquisition illumination measured in situ. This colorimetric image may be tone mapped by state-of-the-art algorithms. We used four tone-mapping techniques later for a representative illustration of visualization experience. We used the code furbished in the Matlab HDR Toolbox [44].

Although the visualization process has the very well defined goal of producing a pleasant and informative visual experience, machine vision output may target several purposes and specific HDR spectral image processing must be considered depending on the task. One particular challenge lies in the best way to handle 32-bit wide pixel information per spectral channel in real time applications. This is also a challenge to compress and store this information, but these aspects are not addressed in this communication.

#### Illumination Constraints on the Pipeline

The role of illumination is major in any imaging system. The first constraint on illumination is that its spectral distribution must be compatible with the camera sensitivities so that it maximizes the signal to noise ratio of the measurement. If there are a priori assumptions on the material surface, it may also be used to tune the illumination. The impact of illumination on the pipeline itself depends highly on the method that is implemented in each of the blocks. First, we assume here that there is no illumination change between the multiple frames that permits multi-exposure. Second, radiance estimation is sensitive to illumination as mentioned in Section 2.6 of Debevec and Malik [21]. for the color case. That means that, if the illumination does not change, there is no issues with the reconstruction of radiance of multiple bands and means also that if there are changes in spectral distribution of the illumination, scaling terms should be adjusted for each of the bands. This is handled in the “Gain adjustment/Channel balance” block of our pipeline. In the article, we measured the light source. We could consider an unknown light, but then the channel balance would benefit from some additional tuning that may be scene dependent. This could be done in several ways: by having a white patch or calibration tile within the scene; by estimating and correct for the illumination based on a priori assumption and statistics of the image [41,42,45]. Demosaicing is not necessarily dependent on illumination, but usually methods based on learning are trained on white balanced images, which makes them sensitive to this aspect. Subsequent color transform and mapping are also dependent on the illumination to guarantee the neutrality of the color image appearance.

## 4. HDR SFA Database

We created a database of images of real scenes, while using this SFA HDR pipeline on the prototype camera defined on Figure 7.

A total of 18 scenes were captured using hardware and conditions shown in Table 1. Care was taken to select three adequate exposure times. A high exposure was selected for which only a few pixels are saturated in low intensities, and a low exposure was selected for which only a few pixels are saturated in high intensities. In the experiment, relatively good highlight conditions were found, and these exposure times were selected to 4, 8 and 16 ms (by doubling the amount of photons hitting the sensors between exposures).

All raw images have been pre-processed to remove the dark noise and the neighboring effects due to NIR blooming, by applying the procedure according to paper [8]. A file set from raw to tone-mapped processed data is available according to the following organization:

- $DB_{0}$: A raw HDR scene with image data stored as single page Tiff files. It contains a set of 8 images (see Figure 5), which are used to reconstruct the camera response (see Figure 6).
- $DB_{1}$: The raw image data for the 18 scenes at the three integration times, stored as single page Tiff files.
- $DB_{2}$: The mosaiced HDR data in .hdr files, which contain raw radiance recovered from the three exposures, following the method by Debevec and Malik [21].
- $DB_{3}$: The demosaiced HDR multispectral images in .mat files, which contain the demosaiced radiances by channel, recovered with the demosaicing algorithm by Miao et al. [43] after that channel sensitivities and illumination are discarded.
- $DB_{4}$: The HDR color CIEXYZ images in .hdr files are computed from the multispectral HDR image by a linear colorimetric calibration computed on the Macbeth ColorChecker.
- $DB_{5}$: The RGB tone mapped .png files for visualization. Four tone-mapping techniques were implemented for comparison, spanning different processing complexities.

Table 2 gives details about the content of the HDR SFA database, including the scene description, the file names and the file extensions. The demosaiced and color transformed HDR images can be, for example, visualized by any software implementation of the PFSTools framework by Mantiuk et al. [46] (e.g., Luminance HDR software). By using these tools, the user could visualize on his screen the 8-bits data per RGB channels, enables a HDR rendering and select a tone-mapping algorithm among several methods to visualize the file. Along with the complete database of images, a Matlab script, named *Script.m* is provided to load data into the workspace. The user can select which scene data to load amongst the 18 scenes available.

## 5. Analysis

### 5.1. Qualitative Evaluation

**Figure 8:**
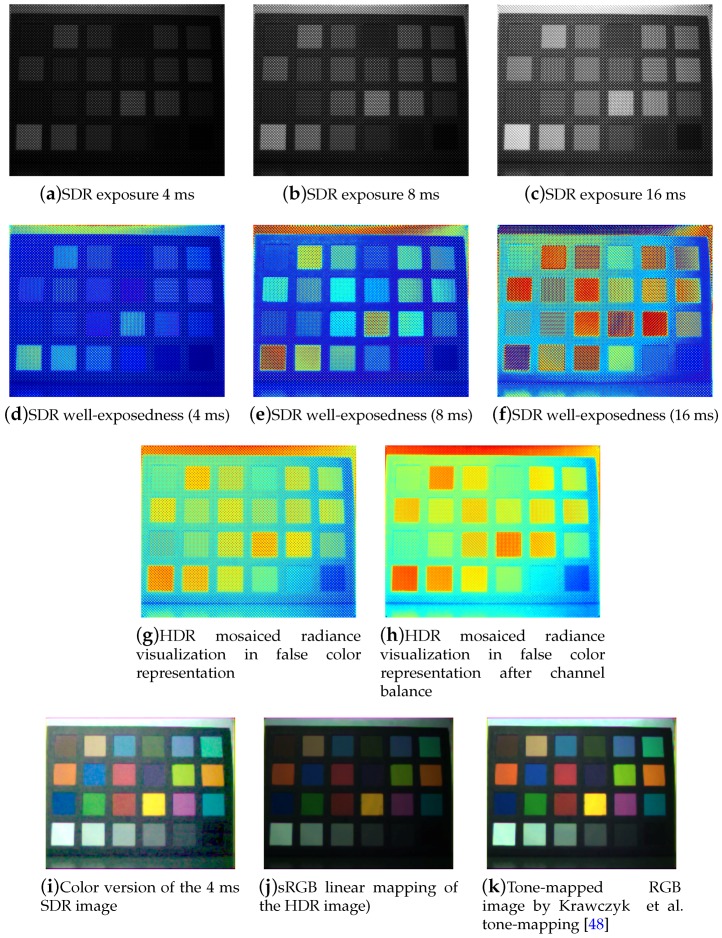
Illustration of the pipeline results for the Macbeth ColorChecker image (a typical low dynamic range scene). (**a**–**c**) Raw images at different exposures; (**d**–**f**) false color well-exposedness representation that use the Jet colormap from MATLAB; (**g**,**h**) HDR radiance mosaiced image estimated from the three exposure set (**a**–**c**) and visualized before and after the channel balance using the Jet colormap from MATLAB; (**i**-**k**) color representation of the image based on the SDR single acquisition or after tone-mapping of the HDR images.

We provide an exhaustive example of images at each step of the pipeline for one scene in Figure 8.

We observe that channels are unevenly affected by noise at the different exposures. This phenomenon is highlighted in Figure 8d–f, where a pixel position could hold a good intensity for a given exposure time (reddish colors of the Jet colormap), and a bad exposition in another (bluish colors). If we look at the raw images of Macbeth ColorChecker, at one neutral patch, we can clearly distinguish the inherent energy balance problems between pixel values through the 8 channels. In the white neutral patch, the NIR channel called $S_{8}$ is saturated in the middle integration time. These problems lead to visual noise when visualizing a single SDR reconstructed image (see Figure 8i). Our HDR-SFA imaging pipeline corrects the problem by a certain amount. On Figure 8g, we can observe the HDR mosaiced image of radiance, that exhibits unbalanced sensitivities by channel. After we applied the balance correction, we observe on Figure 8h that we have a more homogeneous representation of the achromatic patches through the different channels.

The global effect of applying our HDR pipeline can be visually appreciated on the color tone-mapped version of the image on Figure 8i,k.

In terms of scene dynamic range, we know that the Macbeth ColorChecker scene is a typical low dynamic range scene. We could capture the whole dynamic range of the scene with only one exposure (i.e., Figure 8i) and the HDR process used permits only to reduce the noise and to solve the energy balance issue. However, for a higher dynamic range scene (like the CD scene), in addition to balancing the exposure among the pixels, we also extend the dynamic range by a certain amount. This is evaluated below.

**Figure 9:**
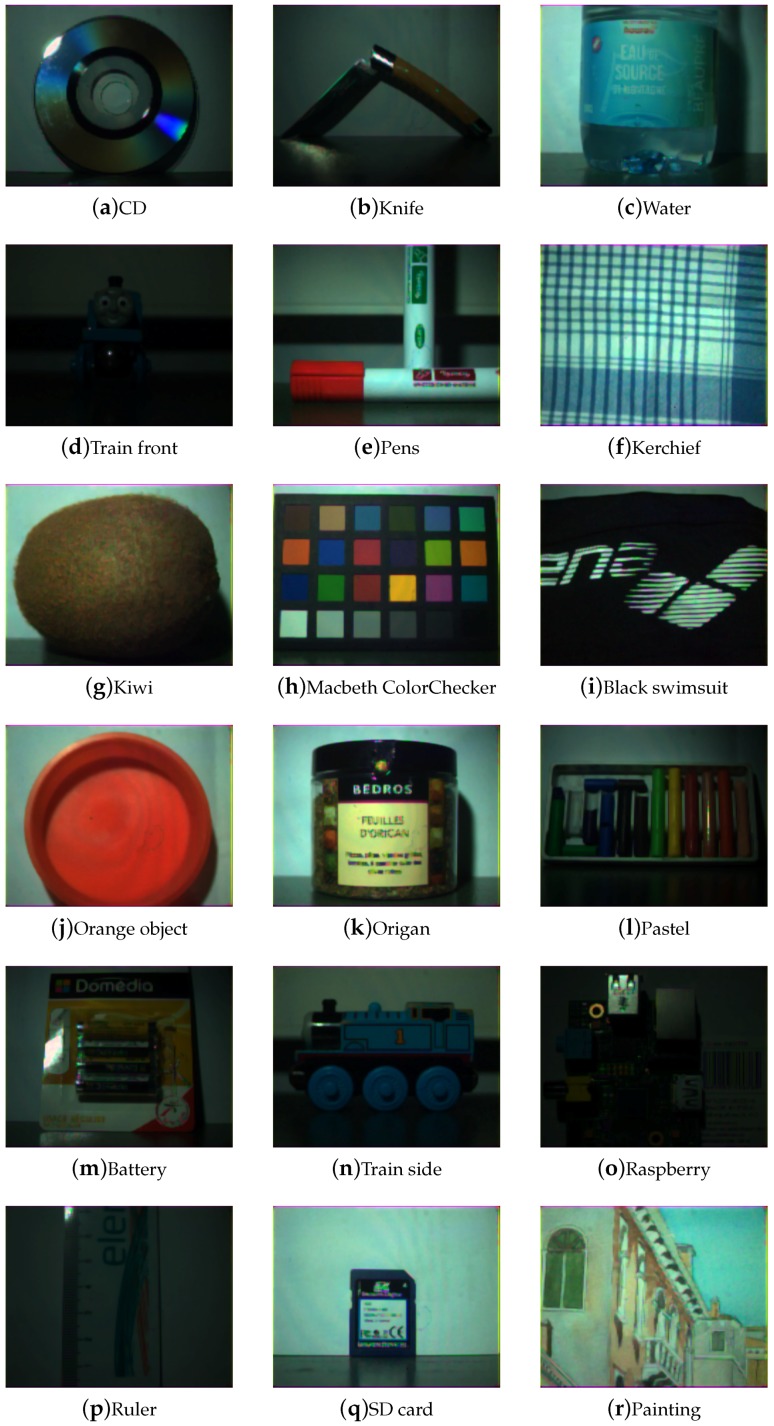
Database visualization of all scenes using a global logarithmic tone-mapping. In case of a high dynamic range scene with high specular reflection, good details are accomplished in specular regions, at the expense of a global image contrast reduction.

**Figure 10:**
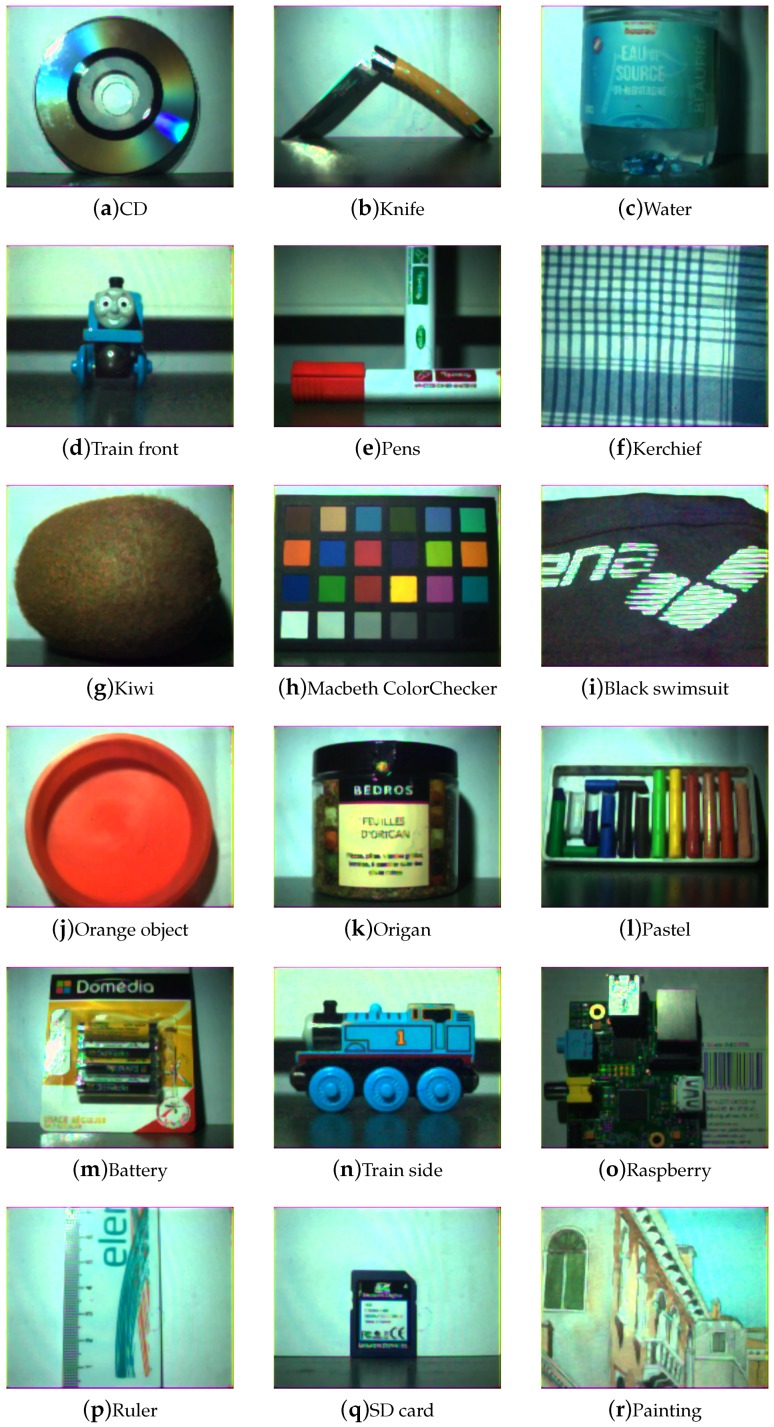
Database visualization of all scenes using a tone-mapping that is a combination of local and global anchoring of brightness values; the Krawczyk et al. [48] tone-mapping. Global contrast is preserved even in the presence of high specular reflections.

**Figure 11:**
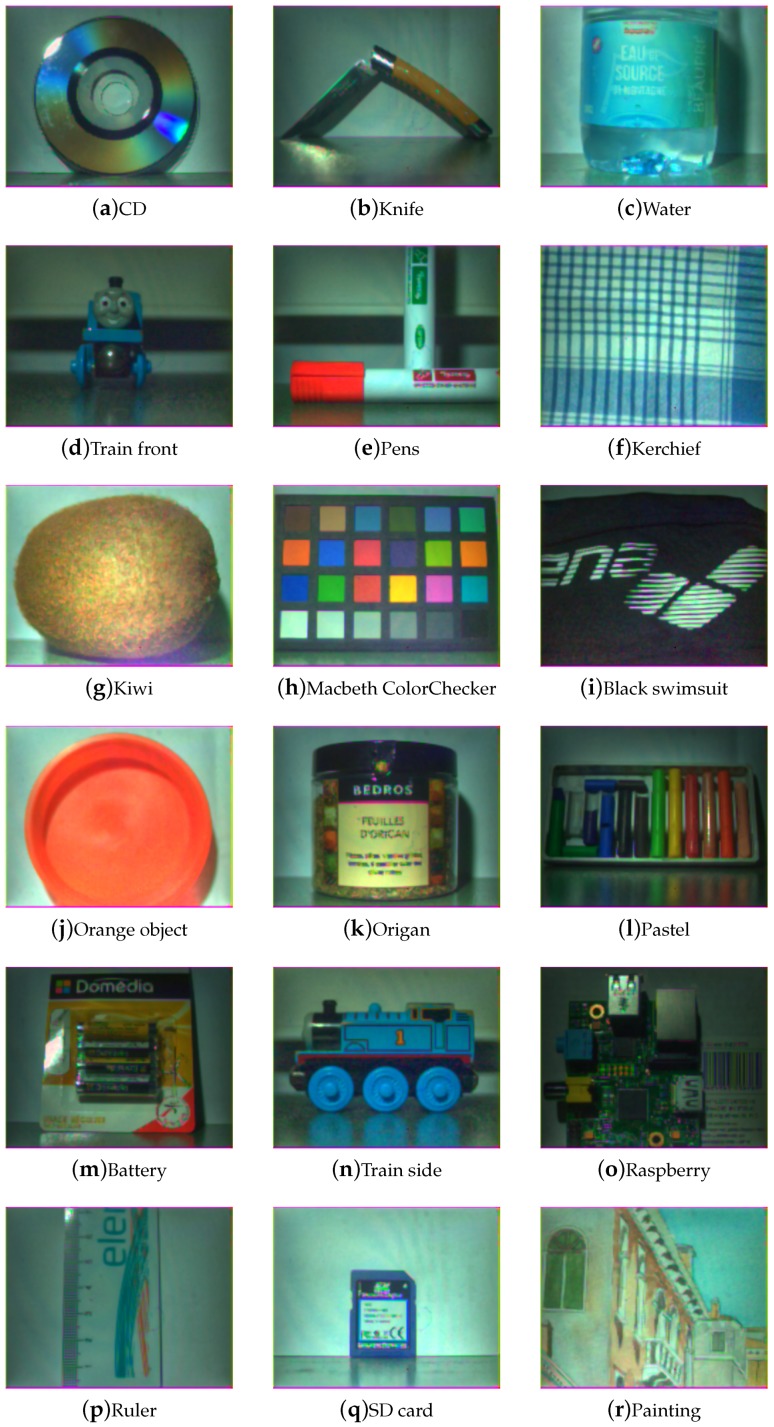
Database visualization of all scenes using the gradient domain compression tone-mapping by Fattal et al. [49]. We can see that this technique highlights details well in areas affected by shadows. It gives good details in specular regions, preserving a relatively good global contrast in the scene.

**Figure 12:**
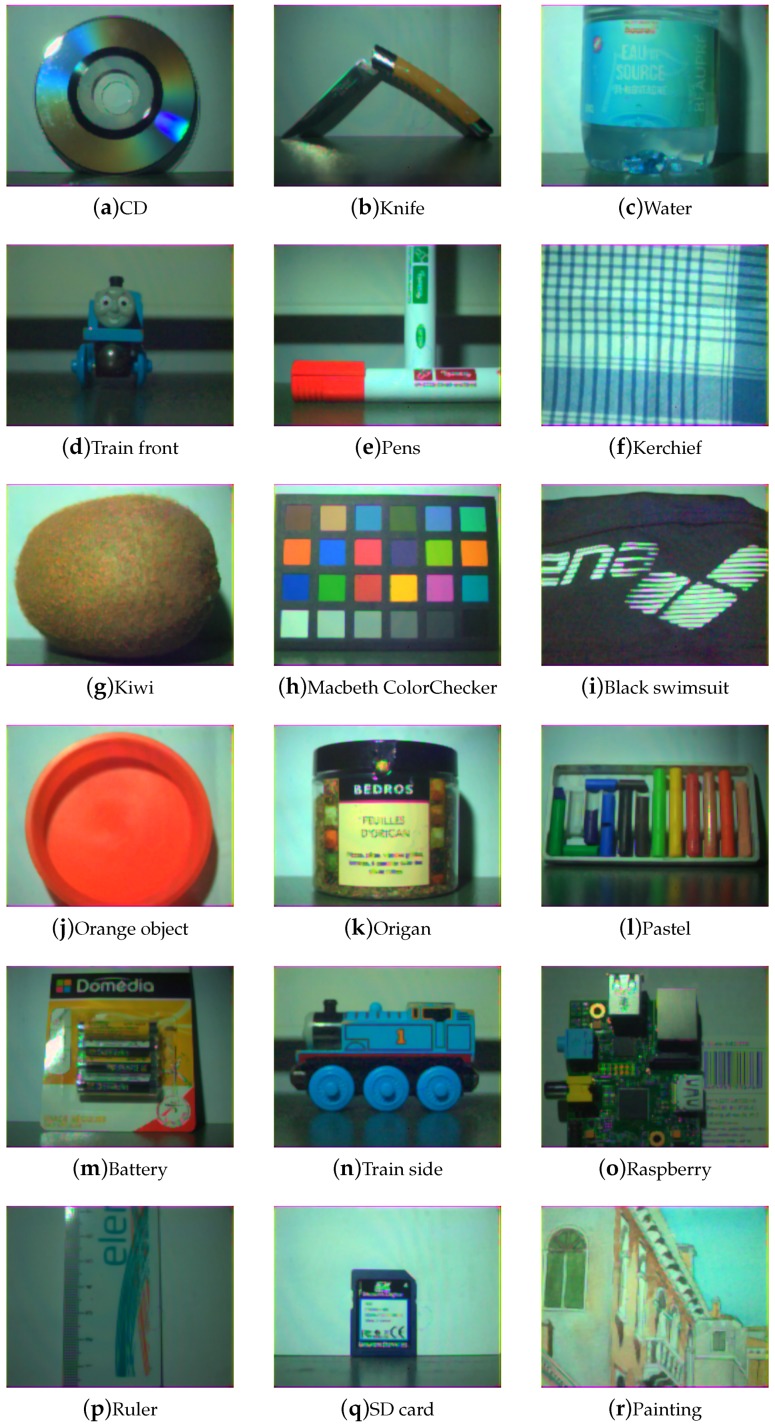
Database visualization of all scenes using a tone-mapping that is a combination of local and global tone-mapping developed by Banterle et al. [50] tone-mapping. We observe that this technique achieves good rendering in term of local and global contrasts.

Figure 9, Figure 10, Figure 11 and Figure 12 show the tone-mapped color images of the database processed with different algorithms. Namely, we applied a simple logarithmic mapping, Krawczyk et al. [48], Fattal et al. [49] and Banterle et al. [50].

**Figure 13:**
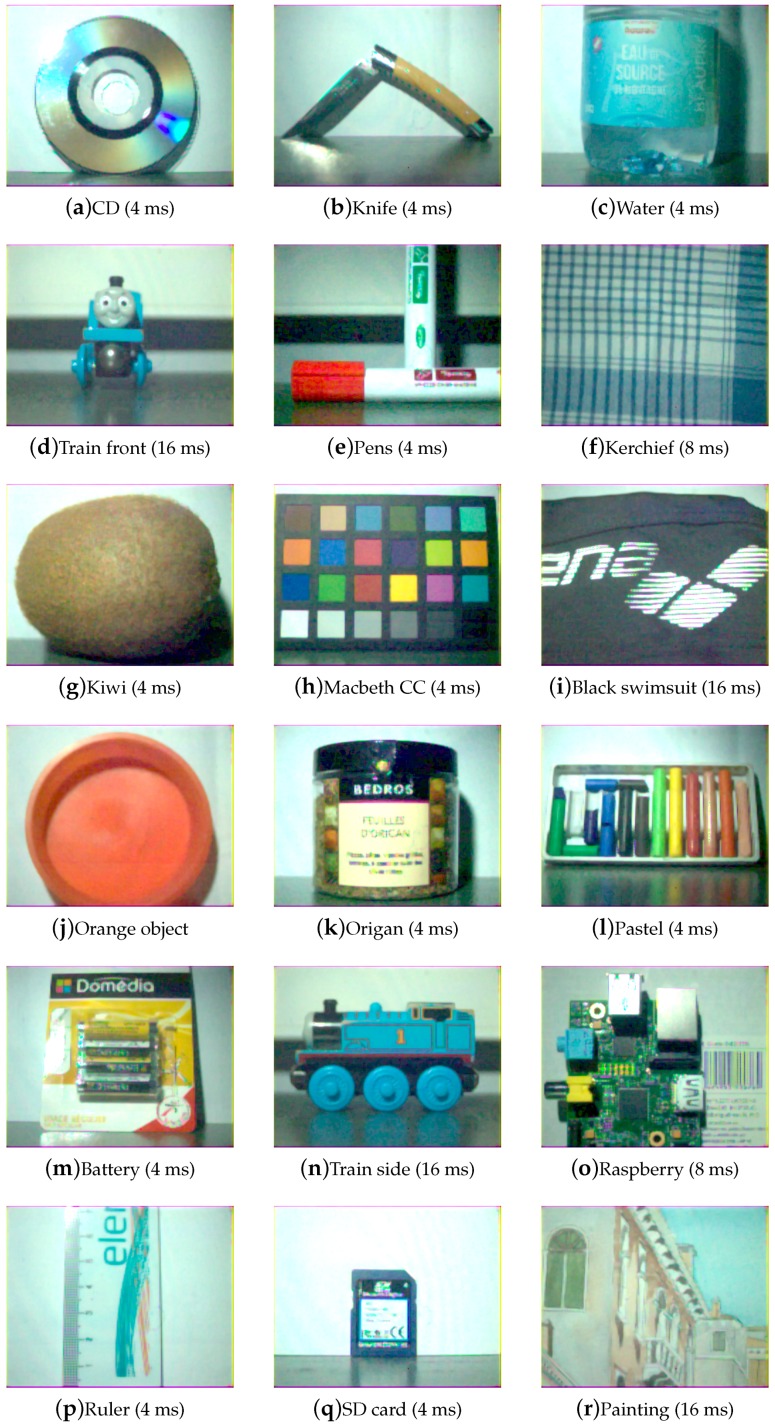
Visualization of SDR color versions of the scenes without using any HDR processing. Integration times was selected to be the best exposure as described in [32]. Those SDR versions of scenes could be compared to the output images of HDR pipeline with tone-mapping.

Global impression is that noise is reduced and that the quality of the images is better than the best SDR versions of these images shown in Figure 13. Difference in the tone-mapping algorithms seems to impact mostly the global brightness of the images, which also depends on the scene. Highlights that are not handled by the shortest integration time are still not handled after the process. This is due to the selected exposure time, which may not be optimal for some scenes with high dynamic range of radiance. However, the quality of the scenes or part of the scenes of low dynamic have been greatly improved, such as in the Macbeth ColorChecker image. Spatial artifacts, such as seen on the SD card image, are due mostly to other optical and sampling effects, which we do not assess in this work (non-uniform illumination, optical effects, and aliasing in demosaicing).

### 5.2. Quantitative Evaluation

We propose to evaluate our pipeline quantitatively by several strategies. Difficulty in this task comes from the fact that no ground truth is available and from the fact that there are a great number of factors that affect the final image quality. We propose to evaluate only two aspects: The radiance estimation and the final tone-mapped color image.

#### 5.2.1. Radiance Estimation

**Figure 14:**
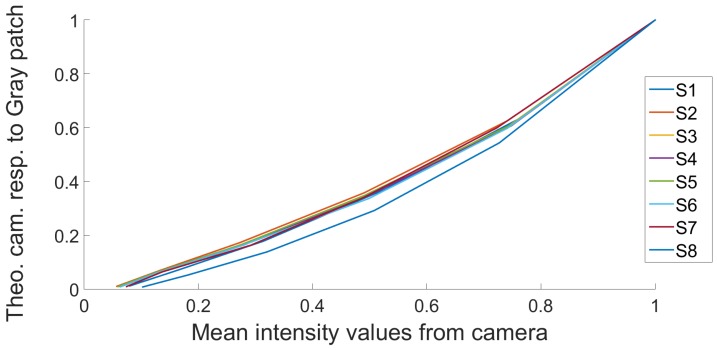
Study of the channel camera response according to the achromatic patches in the Macbeth ColorChecker chart. A theoretical response has been computed, taking into account the illuminant and the camera response (see Figure 7a,c).

We are interested first in evaluating the estimation of the radiance of the scene. We propose three indicators:

- We first investigate the relation between the achromatic patches radiance of the Macbeth ColorChecker evaluated by the camera and computed theoretically by spectral simulation on Figure 14. The curves do not exhibit a very good linear behavior and show an offset. The CRF estimation may be impaired by the very high radiance of the lamp in the scene. However, issue with this evaluation is that it is quite affected by the spatial non-uniformity of the illumination, so it is not easy to draw strong conclusions from it. Indeed, the achromatic patches are distributed horizontally across the image, so vignetting and illumination shift impact the results. The curve of the channel sensitive to the NIR is showing an outlying behavior. This specific channel is not very well evaluated by this indicator due to lack of measurements of the illumination between 1000 and 1100 nm for material limitation (our measurement device did not reach beyond that limit).
- To produce a better evaluation and break the limitation of the above bullet point, we argue that the ratio of intensities, by channel, between patches in simulation and in practice should be the same if the radiance is well evaluated. In addition, if we compute only ratio between adjacent patches, the effect of the illumination and vignetting should be minimum. Difference in ratio *r* between a couple of horizontal adjacent patches is computed such as $\Delta R=\sqrt{{\left({\hat{r}}_{i}^{j}-r_{i}^{j}\right)}^{2}}$, with i∈Nandi∈[1,8] being the channel considered and j∈Nandj∈[1,20], indexing the ratio between each pair considered.
  Results are shown on Table 3. It is shown that we have a minimum and maximum error in the range 0%to46%. This evaluation demonstrates that we obtained a rather good radiance estimate with an average of 5% of error among patch couples and 5% of error among channels. Moreover, the error among channels is near to constant, which indicates that we have a good uniformity in radiance recovery over wavelengths. It appears that the maximum error is reached when couple of patches shows a large difference in intensity values like 4/5, 10/11, 13/14, 15/16. We investigated this point separately and found out that the signal to noise ratio is rather high for specific combinations of radiance and sensor spectral sensitivity. This is due to low radiance from the patch. When the sensor value is high for one patch and very small for another one, this noise is amplified. This creates those huge errors. It is yet to be investigated how this effect impacts the evaluation of HDR radiance accuracy.
- Image quality does not depend only on radiance accurate evaluation, so we also make a tentative to evaluate the process by means of established no-reference metric. This is an attempt to evaluate the global quality of the HDR images. BRISQUE [51] no-reference metric scores are computed per channel on the demosaiced HDR multispectral images. The scores are shown and averaged by channel on the right part of Table 4. The best scores of BRISQUE are close to 0 and the result on the HDR images are closer to 100, which should indicate a very bad quality. However, those results are difficult to interpret since we do not have very natural content, a consistent pixel intensity range, neither any data to compare with. In addition, we did not re-trained BRISQUE for those specific data, so that scores over 100 occurs, meaning that the quality of the image is worse than the training data set wost image quality, according to the measure. Also, HDR linear data may exhibit different statistics than usual gamma corrected SDR images. This is supported by the fact that the magnitude of the score seems not to correlate very well with the observation of the color images (see next paragraph). We observed similar difficulties with scores from NIQE [52] or BLIINDS-II [53] image quality measures. The scores of the two last metrics are not presented in the article because we consider that using those no-reference metrics for this purpose is not adequate. BRISQUE is shown to report an example of this attempt. We cannot state here how BLIINDS-II and BRISQUE would become efficient if they were trained on adequate images of radiance. Nevertheless, we could still observe that the quality of each channel for one image is of similar quality across channels, and since BRISQUE is computed based on image statistics, this indicates that we have an homogeneous quality, which is very good.

#### 5.2.2. Evaluation on Color Images

We are interested in the evaluation of the tone mapped color images to demonstrate that we improved the overall quality. For that, we used again the BRISQUE [51] no-reference metric, and evaluate the results on the left part of Table 4. Red cells with the worst score are highlighted, whereas green cells mean best scores. Results are somehow surprising, since the best observed visual quality is not indicated by the metric (cf. qualitative analysis). We still can explain the worst results by the dark low contrasted images tone-mapped by the global logarithm. Best results of BRISQUE indicates that SDR images are best, that probably satisfy more to the conditions evaluated by the metric. As mentioned above, scores higher than 100 mean that the quality of the image is worse than the set of training images. It seems however that BRISQUE is not the adequate metric to evaluate the quality of tone-mapped images. BLIINDS-II [53] exhibits generally similar results in giving generally the color SDR image as the best between the color images. NIQE [52] does not seem to provide any strong tendency. The scores of the two last metrics are not presented in the article because we estimate that using those no-reference metrics for this purpose is not adequate. BRISQUE is shown to report an example of this attempt.

New metrics are specially designed to evaluate tone-mapped HDR images. We evaluated the different color images by using a state-of-the-art method called HIGRADE [54]. HIGRADE is a dedicated method for non-reference image quality assessment. It evaluates the SDR images obtained by algorithms such as tone-mapping, multi-exposure fusion, or other dedicated processing. This method is dedicated to perceptual evaluation of HDR images and has been considered as the most efficient algorithm on a large database of HDR images, called ESPL-LIVE HDR Image Quality database [55]. Table 5 shows the output of the algorithm for SDR images. This evaluation clearly demonstrates that the HDR versions of the images are better than the SDR one, especially when using the Krawczyk et al. [48] technique. This technique is based on both local and global contrast enhancements, that seems to give a good general rendering, as the metric suggests. Other tone-mapping techniques have relatively good scores, and all provide better scores than the SDR one after averaging. Standard deviation remains quite stable, except for Banterle tone mapping.

### 5.3. Discussion

The implementation of the HDR-SFA pipelines produce images of better visual quality that its SDR counterpart. In the previous section, we made a tentative to evaluate the quality of data which are not easy to handle by quality procedures. First because we do not have references, neither groundtruth or good knowledge on HDR scenes. Indeed, in general HDR images are evaluated visually after being tone-mapped. In our context, it is a little more complex since we also add some spectral dimension to the problem. We try in the following to discuss this according to our evaluation.

One major aspect of SFA images is that we do not have ground truth, for both SDR and HDR domains. For spatial information, we are limited to the result of the demosaicing method or by the fact that the information is sparsely distributed over the whole sensor. For spectral information, we are limited to visual evaluation of content projected in a color space. Evaluation may however be produce on usability on a specific vision task, which is out of the scope of that article.

In our tentative to produce an evaluation on data quality, it appears that we cannot directly compare SDR, HDR and HDR tone-mapped results using a SDR image quality metric (such as BRISQUE). Generally, it is understood that a tone-mapped HDR result tends to compress a high dynamic range of radiance into a displayable range of intensity (e.g., 8-bits), as the logarithmic tone-mapping do. It often leads to an image of rather “washed and gray” appearance and devoid of local and global contrast. In other words, the quantity of visual information is enhanced at the expanse of the naturalness. So evaluation fails when trying to use SDR metrics to evaluate the image quality. That is why dedicated HDR quality metrics, such as HIGRADE [54], have been introduced in the literature, along with emerging perceptual-based tone-mapping techniques [48].

Although we hardly evaluate the radiance estimation, we validated the pipeline for the visualization output by examples and by objective metrics.

Based on this pipeline, we consider two typical types of potential applications. SFA camera are being used in several applications, with promising recent results in, e.g., medical imaging, where the snapshot aspect is rather important to have stability over physiological parameter changes [56]. However, the dynamic of the scene may be very large in such applications (for instance specular reflections on wet tissues or difference of intensity light between outside body and inside an opening in the body). In this case, such pipeline can be useful in situations that are well controlled, including knowledge about the illumination. Evaluation on how three consecutive image captures generate noise for a specific application is yet to be investigated. Temporal corrections of the time-dependent artifacts introduced are well understood in the literature [28]. On the other hand, general computer vision tasks, e.g., background subtraction [57], within uncontrolled illumination, where the dynamic range of the scenes could also be great can be targeted (for instance, automotive car getting out of a tunnel). One of the major constraint in this case is to handle the changes of illumination within the pipeline. Recent works on illuminant estimation from multispectral images [41,42,45] shall permit to implement correction in real time as for camera white balancing. Further works are required to investigate the impact of illumination in those applications. In any application, the HDR imaging pipeline permits to solve issues with energy balance of the sensor, i.e., when two bands have very different sensitivities for a similar integration time.

## 6. Conclusions

We defined a generalized imaging pipeline for HDR-SFA cameras. This is a similar pipeline to CFA architecture, adding spectral processing blocks and HDR enhancement for the channel balance correction and noise reduction. By demonstrating the pipeline, we enable the use of SFA camera in computer vision systems at reduce modification of the existing CFA pipeline.

Further works include the evaluation of the impact of each of the imaging pipeline components with respect to either visualization or usability of the HDR spectral data. We presented one instantiation, while many are possible. Further works also include standardization of camera and pipeline as well as file format and transmission line mixing multiple channel and HDR radiance data.

## Figures and Tables

**Table 1:** Summary of the global parameters and the SFA camera characteristics used during the acquisition.

Camera Sensor	E2V EV76C661 + MSFA-Global Shutter Mode
Camera resolution	1280 × 1024 (sensor native)–319 × 255 (image pre-processed)
Number of bands	8 (7 visible and 1 NIR)
Wavelength (calibrated)	380–1100 nm
Exposure time	3 exposure times: 4–8–16 ms
Illuminant	D65 simulator (see Figure 7a)
Optics/Aperture	Edmund optics 12 mm 58001–F/1.8
Focus	Fixed (20 cm)
Image format	Tiff 8 bits

**Table 2:** The files can be downloaded as supplementary material at http://chic.u-bourgogne.fr [47], where the link SFA_HDR points out to a zip file that contains five directories, one directory for each stage of the pipeline called “DB_#” in Figure 4. The raw SDR, HDR mosaiced, HDR demosaiced, HDR CIEXYZ and RGB color tone mapped data are available to the community for further research.

Database		$DB_{1}$	$DB_{2}$	$DB_{3}$	$DB_{4}$	$DB_{5}$
Scene Name	Dynamic Range	File Name RAW	File Name HDR Mosaiced	File Name HDR Demosaiced	HDR XYZ	HDR Tone Mapped
CD	159.2	raw_preprocessed_cd_"exposure".tiff	hdr_mosaiced_cd.hdr	hdr_demosaiced_cd.mat	hdr_xyz_cd.hdr	hdr_tonemapped_"method"_cd.png
Knife	226.5	raw_preprocessed_knife_"exposure".tiff	hdr_mosaiced_knife.hdr	hdr_demosaiced_knife.mat	hdr_xyz_knife.hdr	hdr_tonemapped_"method"_knife.png
Water	147.3	raw_preprocessed_water_"exposure".tiff	hdr_mosaiced_water.hdr	hdr_demosaiced_water.mat	hdr_xyz_water.hdr	hdr_tonemapped_"method"_water.png
Train front	503.9	raw_preprocessed_train_front_"exposure".tiff	hdr_mosaiced_train_front.hdr	hdr_demosaiced_train_front.mat	hdr_xyz_train_front.hdr	hdr_tonemapped_"method"_train_front.png
Pens	145.6	raw_preprocessed_pens_"exposure".tiff	hdr_mosaiced_pens.hdr	hdr_demosaiced_pens.mat	hdr_xyz_pens.hdr	hdr_tonemapped_"method"_pens.png
Kerchief	78.8	raw_preprocessed_kerchief_"exposure".tiff	hdr_mosaiced_kerchief.hdr	hdr_demosaiced_kerchief.mat	hdr_xyz_kerchief.hdr	hdr_tonemapped_"method"_kerchief.png
Kiwi	216.1	raw_preprocessed_kiwi_"exposure".tiff	hdr_mosaiced_kiwi.hdr	hdr_demosaiced_kiwi.mat	hdr_xyz_kiwi.hdr	hdr_tonemapped_"method"_kiwi.png
Macbeth CC	153.3	raw_preprocessed_macbeth_"exposure".tiff	hdr_mosaiced_macbeth.hdr	hdr_demosaiced_macbeth.mat	hdr_xyz_macbeth.hdr	hdr_tonemapped_"method"_macbeth.png
Black swimsuit	231.4	raw_preprocessed_black_swimsuit_"exposure".tiff	hdr_mosaiced_black_swimsuit.hdr	hdr_demosaiced_black_swimsuit.mat	hdr_xyz_black_swimsuit.hdr	hdr_tonemapped_"method"_black_swimsuit.png
Origan	135.0	raw_preprocessed_origan_"exposure".tiff	hdr_mosaiced_origan.hdr	hdr_demosaiced_origan.mat	hdr_xyz_origan.hdr	hdr_tonemapped_"method"_origan.png
Orange object	42.5	raw_preprocessed_orange_object_"exposure".tiff	hdr_mosaiced_orange_object.hdr	hdr_demosaiced_orange_object.mat	hdr_xyz_orange_object.hdr	hdr_tonemapped_"method"_orange_object.png
Pastel	331.1	raw_preprocessed_pastel_"exposure".tiff	hdr_mosaiced_pastel.hdr	hdr_demosaiced_pastel.mat	hdr_xyz_pastel.hdr	hdr_tonemapped_"method"_pastel.png
Battery	274.7	raw_preprocessed_battery_"exposure".tiff	hdr_mosaiced_battery.hdr	hdr_demosaiced_battery.mat	hdr_xyz_battery.hdr	hdr_tonemapped_"method"_battery.png
Train side	296.6	raw_preprocessed_train_side_"exposure".tiff	hdr_mosaiced_train_side.hdr	hdr_demosaiced_train_side.mat	hdr_xyz_train_side.hdr	hdr_tonemapped_"method"_train_side.png
Raspberry	871.7	raw_preprocessed_raspberry_"exposure".tiff	hdr_mosaiced_raspberry.hdr	hdr_demosaiced_raspberry.mat	hdr_xyz_raspberry.hdr	hdr_tonemapped_"method"_raspberry.png
Ruler	145.6	raw_preprocessed_ruler_"exposure".tiff	hdr_mosaiced_ruler.hdr	hdr_demosaiced_ruler.mat	hdr_xyz_ruler.hdr	hdr_tonemapped_"method"_ruler.png
SD card	72.2	raw_preprocessed_sd_"exposure".tiff	hdr_mosaiced_sd.hdr	hdr_demosaiced_sd.mat	hdr_xyz_sd.hdr	hdr_tonemapped_"method"_sd.png
Painting	130.6	raw_preprocessed_painting_"exposure".tiff	hdr_mosaiced_painting.hdr	hdr_demosaiced_painting.mat	hdr_xyz_painting.hdr	hdr_tonemapped_"method"_painting.png

**Table 3:** Ratio difference between radiance computation and estimation between adjacent patches of the Macbeth ColorChecker. The index refers to the number of the patch on the chart. Except for some specific couple of patches, we could consider a good estimation at less than 5% in average.

-	S1	S2	S3	S4	S5	S6	S7	S8	Mean	STD
1/2	0.01	0.03	0.05	0.03	0.00	0.03	0.02	0.04	0.03	0.02
2/3	0.10	0.09	0.08	0.08	0.05	0.05	0.04	0.09	0.07	0.02
3/4	0.04	0.03	0.02	0.03	0.03	0.03	0.04	0.02	0.03	0.01
4/5	0.46	0.31	0.07	0.08	0.15	0.17	0.23	0.11	0.20	0.03
5/6	0.01	0.03	0.02	0.02	0.00	0.01	0.01	0.02	0.01	0.01
7/8	0.07	0.08	0.06	0.04	0.03	0.02	0.00	0.04	0.04	0.03
8/9	0.06	0.05	0.07	0.08	0.10	0.19	0.23	0.10	0.11	0.07
9/10	0.02	0.02	0.02	0.02	0.02	0.02	0.02	0.03	0.02	0.00
10/11	0.04	0.03	0.09	0.09	0.08	0.06	0.06	0.04	0.06	0.02
11/12	0.02	0.00	0.01	0.01	0.03	0.01	0.03	0.00	0.01	0.01
13/14	0.07	0.04	0.14	0.20	0.20	0.19	0.19	0.12	0.14	0.06
14/15	0.07	0.07	0.03	0.05	0.03	0.02	0.05	0.09	0.05	0.02
15/16	0.05	0.04	0.06	0.07	0.09	0.06	0.05	0.05	0.06	0.02
16/17	0.01	0.03	0.01	0.01	0.00	0.01	0.00	0.01	0.01	0.01
17/18	0.00	0.01	0.04	0.02	0.01	0.00	0.00	0.00	0.01	0.01
19/20	0.06	0.06	0.06	0.06	0.06	0.06	0.05	0.07	0.06	0.01
20/21	0.05	0.05	0.05	0.05	0.04	0.05	0.05	0.06	0.05	0.01
21/22	0.04	0.04	0.04	0.04	0.04	0.03	0.04	0.06	0.04	0.01
22/23	0.04	0.03	0.03	0.03	0.02	0.02	0.01	0.06	0.03	0.01
23/24	0.06	0.04	0.06	0.05	0.03	0.02	0.00	0.09	0.04	0.03
**Mean**	0.06	0.05	0.05	0.05	0.05	0.05	0.06	0.06	-	-
**STD**	0.10	0.06	0.03	0.04	0.05	0.06	0.07	0.04	-	-

**Table 4:** BRISQUE [51] no-reference metric computed on the SDR color images and also on each channel of the multispectral HDR images. Results estimate that the best exposure SDR color image is better than any of the tone-mapped. This is different to what is observed and we may discard BRISQUE to analyze such data. The results on the spectral channels shows very bad BRISQUE scores, but again, they are hardly comparable to anything we know. Nevertheless, they also show that scores are relatively similar across the channels, indicating stability. Red cells with the worst score are highlighted, whereas green cells mean best scores.

Image	SDR	TM	TM	TM	TM	S1	S2	S3	S4	S5	S6	S7	S8
Scene		Banterle	Fattal	Log	Krawczyk								
black_swimsuit	50.2	63.5	72.1	77.5	54.6	88.4	90.4	88.3	92.6	90.2	88.8	78.7	87.5
train_side	29.4	56.8	53.8	65.8	50.7	84.9	85.2	78.8	81.8	87.8	83.4	66.4	89.5
cd	34.0	53.5	53.9	59.7	46.2	54.0	52.5	48.9	47.7	53.5	50.1	44.4	44.2
kiwi	28.4	45.7	39.9	47.0	44.3	46.9	49.3	42.0	42.1	36.5	41.4	32.1	37.3
sd	36.4	50.8	52.0	51.6	47.6	51.7	51.4	49.1	50.8	53.1	56.9	49.5	45.3
pens	18.4	58.1	60.0	63.1	59.3	74.7	76.2	69.0	70.7	73.9	76.0	64.2	75.8
origan	26.9	52.0	50.2	53.1	50.4	55.3	60.7	49.4	57.3	53.1	52.8	49.3	50.3
painting	42.4	45.9	41.2	46.4	39.5	58.6	54.9	52.5	54.2	42.7	51.2	47.7	49.6
macbeth	29.6	61.2	46.1	69.1	66.2	62.0	67.1	63.8	67.5	69.9	69.9	54.4	65.1
knife	32.0	53.3	62.0	58.9	47.9	51.0	49.0	46.4	50.9	54.8	43.4	33.3	44.7
water	24.4	52.4	49.2	53.3	51.9	59.3	56.4	54.3	54.9	52.2	53.8	47.2	53.8
train_front	34.9	60.5	58.4	77.5	51.4	84.1	85.0	77.1	81.6	82.6	79.9	64.2	85.8
kerchief	87.4	102.6	97.5	105.0	105.1	93.6	96.5	98.8	99.0	102.3	99.4	52.9	45.9
pastel	52.1	71.9	59.6	69.7	67.8	70.4	73.5	71.3	69.9	73.9	73.8	68.0	68.3
orange_object	31.5	59.2	49.8	67.5	54.9	60.7	69.9	55.5	59.7	65.8	68.8	56.0	46.5
battery	42.7	56.7	48.4	60.3	49.8	55.0	57.4	55.8	51.3	52.6	49.8	48.7	50.5
raspberry	45.7	59.7	50.7	60.5	56.1	71.0	71.0	63.6	65.1	65.1	66.6	61.8	63.8
ruler	34.5	53.8	49.2	68.3	39.5	44.8	45.6	48.7	47.7	53.1	52.3	46.3	39.3
**Mean**	37.8	58.8	55.2	64.1	54.6	64.8	66.2	61.8	63.6	64.6	64.3	53.6	58.0
**STD**	15.2	12.6	13.0	13.7	14.7	15.0	15.6	15.8	16.3	17.7	16.6	12.0	17.0

**Table 5:** HIGRADE [54] results for evaluation of color images. Scores indicate that the HDR tone-mapped color images are always better than the SDR single exposure version. Scores indicate also that Krawczyk et al. tone-mapping provide best results amongst the tested algorithms, which is also supported qualitatively by visualization of the images.

Image	SDR	TM Banterle	TM Fattal	TM Log	TM Krawczyk
black_swimsuit	−1.33	−0.84	−0.92	−0.91	−0.57
train_side	−0.79	−0.94	−0.96	−1.19	−0.71
cd	−0.72	−0.12	−0.09	−0.93	−0.08
kiwi	−1.07	−0.86	−1.02	−0.62	−0.45
sd	−1.31	−0.39	−0.13	−0.45	−0.47
pens	−1.23	−0.54	−0.70	−0.56	−0.46
origan	−0.90	−0.41	−0.44	−0.13	−0.14
painting	−0.60	−0.64	−0.84	−0.47	−0.43
macbeth	−0.90	−0.50	−0.42	−0.45	0.02
knife	−0.79	−0.39	−0.49	−0.87	−0.07
water	−1.01	−0.92	−0.80	−0.86	−0.70
train_front	−0.95	−0.64	−0.66	−1.18	−0.72
kerchief	−2.02	−2.05	−1.53	−1.77	−1.46
pastel	−1.13	−0.59	−0.55	−0.73	−0.32
orange_object	−0.99	−0.96	−0.42	−0.61	−0.73
battery	−0.92	−0.34	−0.38	−0.79	−0.14
raspberry	−0.95	−0.81	−0.89	−0.93	−0.24
ruler	−0.40	−0.74	−0.77	−1.21	0.02
**Mean**	−1.00	−0.70	−0.67	−0.81	−0.42
**STD**	0.34	0.40	0.34	0.36	0.36
